# Pyrrole-Based Ti(III) and Ti(IV) PNP Pincer Complexes: Insertion of Ketones into the Ti(IV)-Phosphorus Bond

**DOI:** 10.1021/acs.organomet.3c00327

**Published:** 2023-10-05

**Authors:** Gerald Tomsu, Berthold Stöger, Karl Kirchner

**Affiliations:** † Institute of Applied Synthetic Chemistry, TU Wien, Getreidemarkt 9/163-AC, A-1060 Wien, Austria; ‡ X-ray Center, TU Wien, Getreidemarkt 9/163, A-1060 Wien, Austria

## Abstract

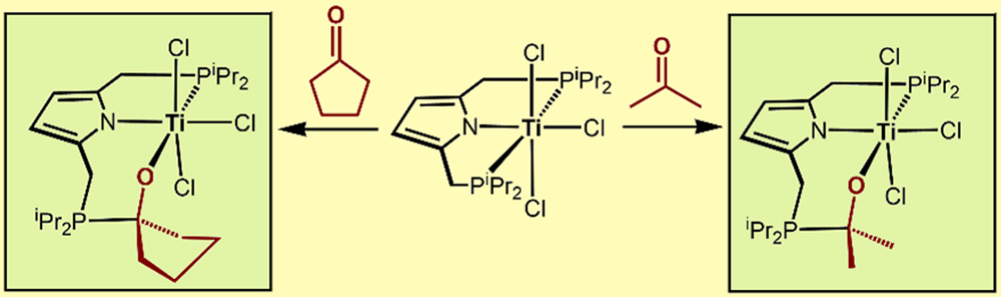

The synthesis, characterization, and reactivity of pyrrole-based Ti(III) and Ti(IV) PNP pincer complexes are described. [P(NH)P-*i*Pr] (**1**) reacts with [TiCl_4_(THF)_2_] at room temperature in the presence of NEt_3_ to afford the Ti(IV) complex [Ti(PNP^*i*Pr^)(Cl)_3_]. This complex reacts with acetone and cyclopentanone to give complexes [Ti(PNO^acet^-*i*Pr)(Cl)_3_] and [Ti(PNO^cyclo^-*i*Pr)(Cl)_3_], respectively. Insertion of the ketone into the Ti(IV)-P bond took place, forming a new tridendate PNO-ligand. Treatment of [TiCl_3_(THF)_3_] with the lithium salt of [P(NH)P-*i*Pr] afforded, upon workup, complex [Ti(PNP-*i*Pr)(Cl)_2_(THF)], a paramagnetic complex with an μ_eff_ value of 1.8(1) μ_B_ which corresponds to one unpaired electron and a formal oxidation state of +III. This compound does not react with ketones. A mechanistic proposal based on DFT calculations is presented. Ketone insertion proceeds via an associative reaction initiated by ketone coordination at the metal center, followed by the opening of the five-membered chelate ring, and finally an intramolecular nucleophilic attack of the noncoordinated phosphine arm at the carbonyl atom of the ketone. All complexes were characterized by X-ray crystallography.

## Introduction

Pincer ligands, which feature an aromatic anionic arene backbone connected to phosphine donors via CH_2_, O, or NR (R=H, alkyl, aryl) linkers and a metal–carbon single bond, so-called PCP pincer ligands, are a very important class of compounds.^1,2^ The simple modification of electronic, steric, and even stereochemical parameters allows the generation of very stable complexes, which are often highly active catalysts for a range of chemical transformations with high selectivity. The vast majority of research utilizing pincer complexes has focused on late-transition metals. Research on pincer complexes of early transition metals, particularly those that involve phosphine donors, is well known.^3^ On the other hand, pincer ligands based on a central anionic pyrrole moiety have received less attention, in particular, when compared to those containing an aryl backbone.^4^ With respect to titanium, pyrrole-based PNP pincer complexes are rare.^5^ Herein, we report on the synthesis, characterization and reactivity of pyrrole-based Ti(III) and Ti(IV) PNP pincer complexes.

## Results and Discussion

**Scheme 1:**
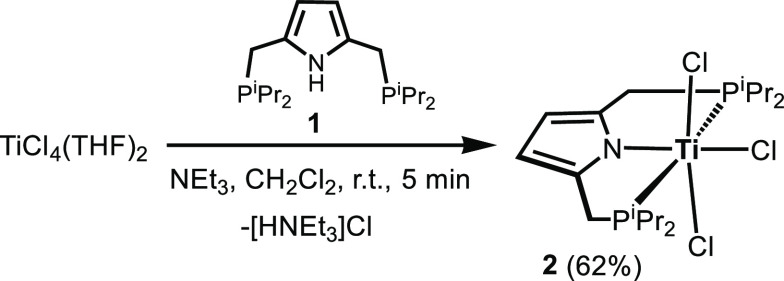
Synthesis of [Ti(PNP-*i*Pr)(Cl)_3_] (**2**)

**Figure 1:**
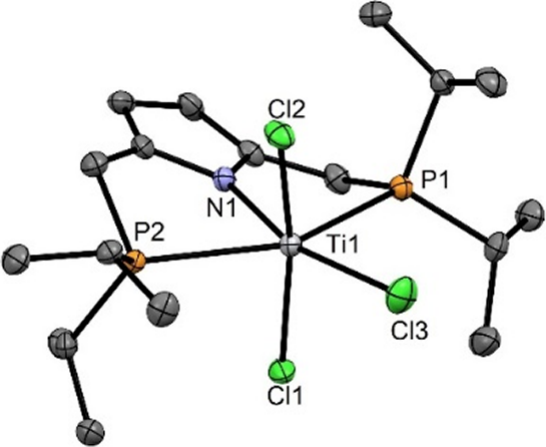
Structural view of [Ti(PNP-*i*Pr)(Cl_3_)] (**2**) showing 50% thermal ellipsoids (H atoms omitted for clarity). Selected bond lengths (Å) and bond angles (deg): Ti1–N1 2.064(3), Ti1–Cl1 2.289(1), Ti1–Cl2 2.310(1), Ti1–Cl3 2.298(1), Ti1–P1 2.577(1), Ti1–P2 2.596(1), N1–Ti1–Cl3 160.6(1), Cl1–Ti1–Cl2 165.2(1), P1–Ti1–P2 149.2(1).

The reaction of [P(NH)P-*i*Pr] (**1**) with [TiCl_4_(THF)_2_] in CH_2_Cl_2_ at room temperature (RT) in the presence of NEt_3_ leads to the formation of the Ti(IV) complex [Ti(PNP^*i*Pr^)(Cl)_3_] (**2**) (Scheme 1). This complex was isolated as a dark brown solid in 62% yield. Characterization was accomplished by a combination of ^1^H, ^13^C{^1^H}, and ^31^P{^1^H} NMR spectroscopy and elemental analysis. Additionally, complex **2** was characterized by X-ray crystallography. A structural view is shown in Figure 1 with selected bond distances and angles reported in the caption.

The coordination geometry around the titanium center is best described as antiprismatic and is similar to that observed in the previously reported structure of 2,6-bis[(dimethylamino)methyl]phenyltitanium(IV) trichloride, a Ti(IV) NCN pincer complex.^6^ The Cl1–Ti1–Cl2, N1–Ti–Cl3, and P1–Ti–P2 angles deviate significantly from 180°, being 165.2(1)°, 160.6(1)°, and 149.2(1)°, respectively.

**Scheme 2:**
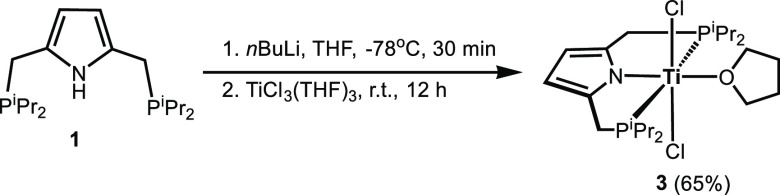
Synthesis of [Ti(PNP-*i*Pr)(Cl)_2_(THF)] (**3**)

Treatment of [TiCl_3_(THF)_3_] with the lithium salt of [P(NH)P-*i*Pr] (**1**), prepared in situ by reacting **1** with *n*BuLi at −78 °C in THF, at RT for 12 h afforded, upon workup, complex [Ti(PNP-*i*Pr)(Cl)_2_(THF)] (**3**) in 65% isolated yield (Scheme 2). A similar complex was reported by Nishibayashi and co-workers with PNP-*t*Bu resulting in the formation of [Ti(PNP-*t*Bu)(Cl)_2_]. In contrast to **3** no THF coordinates to the titanium center, presumably due to the more steric demanding *t*Bu substituents at the phosphorus atoms.^5^ Complex **3** is paramagnetic. A measurement of the magnetic susceptibility in solution (Evans method,^7^ CH_2_Cl_2_) gave an μ_eff_ value of 1.8(1) μ_B_ which corresponds to one unpaired electron and a formal oxidation state of +III. The solution magnetic moment of **3** is comparable to that of [Ti(PCP-*t*Bu)(Cl)_2_] and [Ti(PNP-*t*Bu)(Cl)_2_] being 1.57 and 1.60 μ_B_, respectively.^3,5^

**Figure 2:**
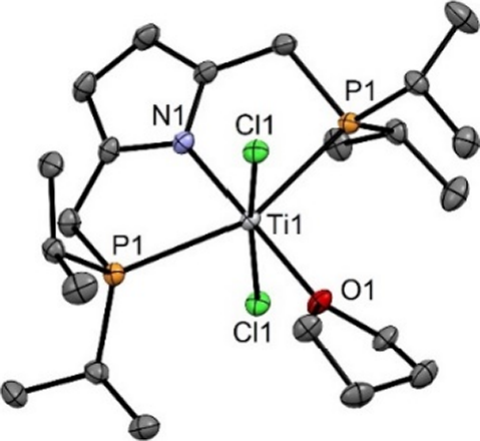
Structural view of [Ti(PNP-*i*Pr)(Cl_2_)(THF)] (**3**) showing 50% thermal ellipsoids (H atoms omitted for clarity). Selected bond lengths (Å) and bond angles (deg): Ti1–N1 2.058(2), Ti1–O1 2.187(2), Ti1–Cl1 2.3481(5), Ti1–P1 2.6220(5), N1–Ti1–O1 180.0, Cl1 Ti1–Cl1–169.88(2), P1–Ti1–P1 155.31(2).

The molecular structure of **3** was determined by X-ray crystallography. A structural view is depicted in Figure 2 with selected bond distances given in the captions. The coordination geometry around the titanium center corresponds to a slightly distorted octahedron where the PNP ligand and THF molecule define the equatorial plane and the two chloride ligands the axial positions.

**Scheme 3:**
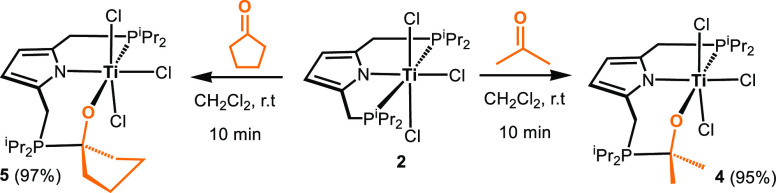
Insertion of Acetone and Cyclopentanone into the Ti–P bond of [Ti(PNP-*i*Pr)(Cl)_3_] (**2**)

**Figure 3:**
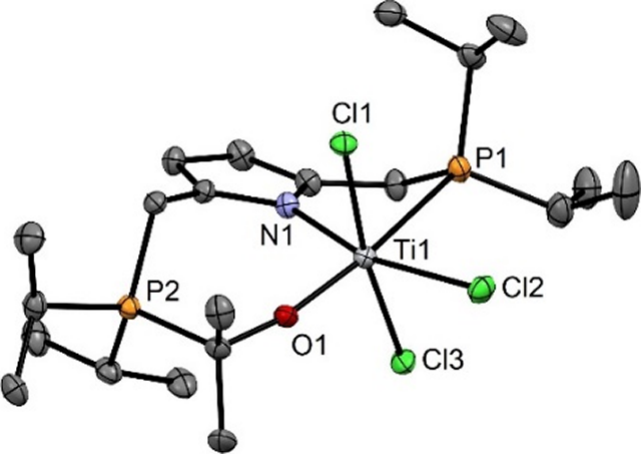
Structural view of [Ti(PNO^acet^-*i*Pr)(Cl)_3_] (**4**) showing 50% thermal ellipsoids (H atoms omitted for clarity). Selected bond lengths (Å) and bond angles (°): Ti1–O1 1.778(2), Ti1–N1 2.064(2), Ti1–Cl1 2.369(1), Ti1–Cl2 2.3604(9), Ti1–Cl3 2.3733(9), Ti1–P1 2.6597(9), N1–Ti1–Cl2 162.90(7), O1–Ti1–P1 165.78(7), Cl1–Ti1–Cl3 172.19(4).

**Figure 4:**
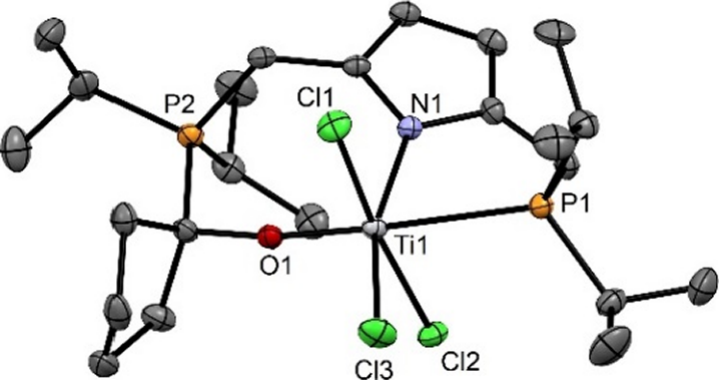
Structural view of [Ti(PNO^cyclo^-*i*Pr)(Cl)_3_] (**5**) showing 50% thermal ellipsoids (H atoms omitted for clarity). Selected bond lengths (Å) and bond angles (°): Ti1–O1 1.773(2), Ti1–N1 2.060(2), Ti1–Cl1 2.360(1), Ti1–Cl2 2.391(1), Ti1–Cl3 2.354(1), Ti1–P1 2.641(1), O1–Ti1–P1 165.89(8), Cl1–Ti1–Cl2 172.09(4), N1–Ti1–Cl3 161.82(8).

Treatment of **2** with 1 equiv of acetone or cyclopentanone in CH_2_Cl_2_ at RT for 10 min afforded, after workup, [Ti(PNO^acet^-*i*Pr)(Cl)_3_] (**4**) and [Ti(PNO^cyclo^-*i*Pr)(Cl)_3_] (**5**), respectively, in 95 and 97% isolated yields (Scheme 3). Insertion of the ketones into the Ti(IV)-P bond took place, forming a new tridendate PNO-ligand. It has to be noted that aldehydes did not react with **2**. A related reaction where ferrocene carboxaldehyde was inserted into the Ti–P bond of the titanocene cation [Cp_2_Ti(C(Ph)=C(Ph)PCy_2_]^+^ was reported.^8^ Moreover, cationic zirconocene phosphinoaryloxide complexes were shown to insert CO_2_ into the Zr(IV)-P bond.^9^ Complexes **3** and **4** are pale red solids, which were characterized by ^1^H, ^13^C{^1^H}, and ^31^P{^1^H} NMR spectroscopy and elemental analysis. Additionally, both complexes were characterized by X-ray crystallography. Structural views are shown in Figures 3 and 4 with selected bond distances and angles reported in the captions. It has to be noted that, in contrast to complex **2**, complex **3** does not undergo insertion reactions with ketones. We assume that THF is bound too strongly to the Ti(III) center, preventing its replacement by ketones.

In the ^31^P{^1^H} NMR spectrum, two phosphorus signals are observed as two singlets at 40.9 and 25.2 ppm for **4** and 35.4 and 31.8 ppm for **5** (cf. complex **2** exhibits one singlet at 81.4 ppm). The ^1^H NMR spectrum of **4** exhibits two singlets at 1.88 and 1.86 ppm assignable to the two inequivalent CH_3_ groups of the inserted acetone.

Crystals suitable for X-ray diffraction for both complexes were obtained by layering a saturated CH_2_Cl_2_ solution of **4** or **5**, respectively, with *n*-pentane. The molecular structures unambiguously reveal that insertion of the ketones into the Ti–P bond took place. Both complexes have a distorted octahedral geometry with bond angles of 162.90(7)° (N1–Ti1–Cl2), 172.19(4)° (Cl1–Ti1–Cl3), and 165.78(7)° (P1–Ti1–O1) for **4** and of 161.82(8)° (N1–Ti1–Cl3), 172.09(4)° (Cl1–Ti1–Cl2), and 165.89(8)° (P1–Ti1–O1) for **5**. The PNO-ligand adopts a meridional geometry. The C–O bond distances for **4** and **5** are 1.406(3) and 1.411(4) Å, respectively, and correspond to a single bond.

**Figure 5:**
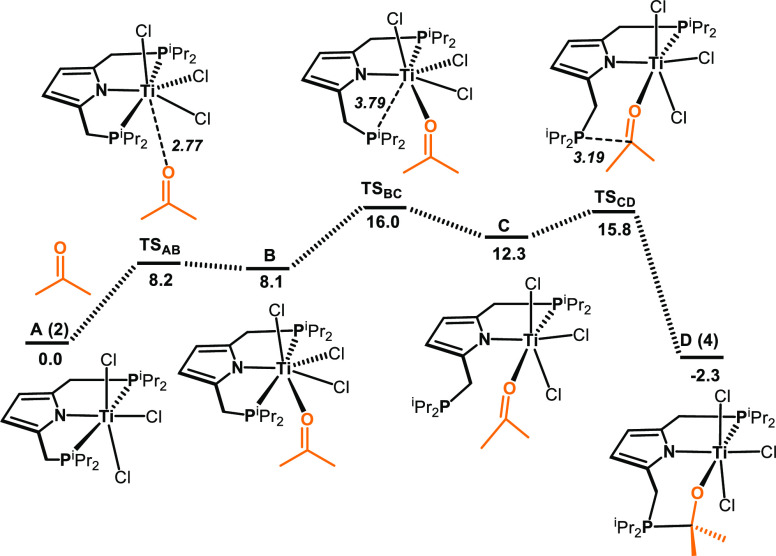
Free energy profile calculated for the for the insertion of acetone into the Ti–P bond of [Ti(PNP-*i*Pr)(Cl)_3_] (**2**) (**A** in the calculations) via an associative pathway. Free Energies (kcal/mol) are referred to **A**. Bond distances (Å) are in italic.

A reaction pathway for the insertion of acetone into the Ti–P bond of [Ti(PNP-*i*Pr)(Cl)_3_] (**2**) (**A** in the calculations) was investigated by DFT calculations. A free energy profile for this reaction is presented in Figure 5. The mechanism obtained starts with the addition of acetone to **A** leading to the seven-coordinated intermediate **B**. Noteworthy, seven coordinate Ti(IV) complexes were reported in the literature.^10^ This reaction is endergonic (Δ*G* = 8.1 kcal/mol) with a small barrier of 8.2 kcal/mol. In the next step, the opening of the five-membered chelate ring takes place to give intermediate **C** with a free energy of activation of 7.9 kcal/mol. The noncoordinated phosphine finally attacks the carbonyl carbon atom of the coordinated acetone to form complex **D**. This intramolecular nucleophilic attack is rather facile, with a barrier of merely 3.5 kcal/mol. The overall process, in agreement with the experiment, is slightly exergonic by 2.3 kcal/mol.

An alternative dissociative pathway involving phosphine arm hemilability and Ti–P bond cleavage was also considered, but was found to be less favorable with a barrier of 17.9 kcal/mol (cf. **TS**_**AB**_ being 8.2 kcal/mol for the associative pathway shown in Figure 5).

## Conclusions

In sum, we prepared the Ti(IV) and Ti(III) PNP pincer complexes [Ti(PNP^*i*Pr^)(Cl)_3_] (**2**) and [Ti(PNP-*i*Pr)(Cl)_2_(THF)] (**3**) from the pyrrole-based ligand precursor [P(NH)P-*i*Pr] (2,5-Bis[[bis(1-methylethyl)phosphino]methyl]-1*H*-pyrrole) (**1**). Complex **2** reacts rapidly with acetone and cyclopentanone to undergo an insertion into the Ti–P bond to yield the nonsymmetric complexes [Ti(PNO^acet^-*i*Pr)(Cl)_3_] (**4**) and [Ti(PNO^cyclo^-*i*Pr)(Cl)_3_] (**5**), respectively, featuring new tridendate PNO-ligands. The Ti(III) complex **3** did not react with ketones. Computational mechanistic studies revealed that ketone insertion proceeds via an associative reaction initiated by ketone coordination at the metal center, followed by the opening of the five-membered chelate ring, and finally with an intramolecular nucleophilic attack of the noncoordinated phosphine arm at the carbonyl atom of the ketone. The overall process, in agreement with experimental data, is very facile with the highest barrier being 16.0 kcal/mol and is exergonic by 2.3 kcal/mol. The structures of all compounds were established by X-ray crystal structure determinations.

## Experimental Section

### General Information

All manipulations were performed under an inert atmosphere of argon by using Schlenk techniques or in an MBraun inert-gas glovebox. The solvents were purified according to standard procedures.^11^ The deuterated solvents were purchased from Eurisotop SAS and dried over 4 Å molecular sieves. The ligand precursor [P(NH)P-*i*Pr] (**1**) (2,5-Bis[[bis(1-methylethyl)phosphino]methyl]-1*H*-pyrrole) was prepared according to the literature.^12^ All other starting materials are known compounds and were used as obtained from commercial sources. ^1^H, ^13^C{^1^H}, and ^31^P{^1^H} NMR spectra were recorded on Bruker AVANCE-250, AVANCE-400, and AVANCE-600 spectrometers. ^1^H and ^13^C{^1^H} NMR spectra were referenced internally to residual protio-solvent and solvent resonances, respectively, and are reported relative to tetramethylsilane (δ = 0 ppm). ^31^P{^1^H} NMR spectra were referenced externally to H_3_PO_4_ (85%) (δ = 0 ppm).

### Synthesis of [Ti(PNP-*i*Pr)(Cl)_3_] (**2**)

[P(NH)P-*i*Pr] (**1**) (0.5 g, 1.53 mmol) was dissolved in CH_2_Cl_2_ (5 mL). TEA (0.212 mL, 1.53 mmol) and [TiCl_4_(THF)_2_] (0.484 g, 1.45 mmol, 0.95 equiv) were added to the solution. After 5 min, the solvent was evaporated, and the residue was dissolved in toluene and filtered over celite. After evaporation of the solvent, the dark brown residue was washed with *n*-pentane (2 × 15 mL). The complex was isolated as a dark brown powder. Yield: 0.454 g (62%). Crystals suitable for an X-ray diffraction study were obtained from a saturated solution of the complex in CH_2_Cl_2_ layered with *n*-pentane. ^1^H NMR (δ, CD_2_Cl_2_, 20 °C): 5.69 (s, 2H, Pyr^3,4^), 3.41–3.28 (m, 4H, CH_2_), 2.48–2.41 (m, 4H, C*H*CH_3_), 1.32–1.26 (m, 26H, CHC*H*_3_). ^13^C NMR (δ, CD_2_Cl_2_, 20 °C): 141.3 (vt, *J* = 6.2 Hz, Pyr^2,5^), 105.6 (vt, *J* = 4.2 Hz, PyrH^3,4^), 27.7 (vt, *J* = 6.3 Hz, *C*HCH_3_), 27.2 (vt, *J* = 10.5 Hz, *C*H_2_), 19.4 (CH*C*H_3_), 19.2 (CH*C*H_3_). ^31^P{^1^H} NMR (δ, CD_2_Cl_2_, 20 °C): 81.4. Anal. Calcd for C_18_H_34_Cl_3_NP_2_Ti: C, 44.98; H, 7.13; N, 2.91. Found: C, 45.15; H, 7.00; N, 2.96.

### Synthesis of [Ti(PNP-*i*Pr)(Cl)_2_(THF)] (3)

A solution of [P(NH)P-*i*Pr] (**1**) (0.2 g, 0.61 mmol) in THF (8 mL) was treated with *n*BuLi (0.382 mL, 1.6 M in hexane, 0.61 mmol) at −78 °C. After stirring for 30 min at this temperature and 30 min at RT, TiCl_3_(THF)_3_ (204 mg, 0.54 mmol) was added. The reaction was stirred for 12 h at RT. All volatiles were removed under reduced pressure. The residue was resolved in toluene (5 mL) and filtered through a pad of celite and solvent was evaporated. By washing the residue twice with *n*-pentane (10 mL), the product was obtained as pink powder. Yield: 210 mg (65%). Single crystals for X-ray diffraction measurement were obtained by layering a saturated CH_2_Cl_2_ solution with *n*-pentane. μ_eff_ = 1.8(1) μ_B_ (CH_2_Cl_2_, Evans method). Anal. Calcd for C_22_H_42_Cl_2_NOP_2_Ti: C, 51.08; H, 8.18; N, 2.71. Found: C, 51.25; H, 8.01; N, 2.61.

### Synthesis of [Ti(PNO^acet^-*i*Pr)(Cl)_3_] (**4**)

[Ti(PNP-*i*Pr)(Cl)_3_] (**2**) (15 mg, 0.00312 mmol) was dissolved in CH_2_Cl_2_ (2 mL) and acetone (2.3 μL, 0.00312 mmol) was added. After 10 min, the solvent was evaporated, and the residue was washed with *n*-pentane (2 × 10 mL). The product was isolated as light brown powder. Yield: 0.016 mg (95%). Crystals suitable for an X-ray diffraction study were obtained from a saturated solution of the complex in CH_2_Cl_2_ layered with *n*-pentane. ^1^H NMR (δ, CD_2_Cl_2_, 20 °C):5.82 (bs, 1H, Pyr^3,4^), 5.67 (bs, 1H, Pyr^3,4^), 4.18 (bs, 1H, CH_2_), 3.88 (bs, 1H, CH_2_), 3.21 (d, *J* = 7.2 Hz, 2H, CH_2_), 2.81–2.61 (m, 3H, C*H*CH_3_), 2.56–2.40 (m, 1H, C*H*CH_3_), 1.87 (d, *J* = 13.0 Hz, 6H, CH_3_CCH_3_)1.47–1.42 (m, 12 H, C*H*_*3*_CHC*H*_*3*_), 1.33–1.28 (m, 12H, C*H*_*3*_CHC*H*_*3*_). ^13^C NMR (δ, CD_2_Cl_2_, 20 °C): 141.0 (vdd, *J* = 7.8, 3.7 Hz, Pyr^2,5^), 125.4 (vdd, *J* = 10.7, 2.8 Hz, Pyr^2,5^), 111.6 (vd, *J* = 8.5 Hz, Pyr^3,4^), 103.3 (vdd, *J* = 6.9, 2.8 Hz, Pyr^3,4^), 88.6 (vdd, *J* = 46.3, 8.8 Hz, CH_3_*C*CH_3_), 28.6 (vd, *J* = 5.1 Hz, *C*H_3_C*C*H_3_), 26.8 (vd, *J* = 17.3 Hz, CH_2_P), 26.0 (vd, *J* = 8.4 Hz, *C*HP^+^), 22.7 (vd, *J* = 32.8 Hz, *C*HP), 19.8 *C*H_3_CH*C*H_3_, CH_2_P^+^), 18.0 (vd, *J* = 3.8 Hz, *C*H_3_CH*C*H_3_, CH_2_P^+^), 17.5 (vd, *J* = 3.6 Hz, *C*H_3_CH*C*H_3_, CH_2_P^+^). ^31^P{^1^H} NMR (δ, CD_2_Cl_2_, 20 °C): 40.9, 35.2. Anal. Calcd for C_21_H_40_Cl_3_NOP_2_Ti. Found: C, 46.82; H, 7.48; N 2.60. Found: C, 46.88; H, 7.58; N, 2.70.

### Synthesis of [Ti(PNO^cyclo^-*i*Pr)(Cl)_3_] (**5**)

[Ti(PNP-*i*Pr)(Cl)_3_] (**2**) (15 mg, 0.00312 mmol) was dissolved in CH_2_Cl_2_ (2 mL) and cyclopentanone stored over molecular sieve (3.0 μL, 0.00343 mmol, 1.1 equiv) was added. After 10 min, the solvent was evaporated, and the residue was washed with *n*-pentane (2 × 10 mL). The product was isolated as light brown powder. Yield: 0.017 mg (97%). Crystals suitable for an X-ray diffraction study were obtained from a saturated solution of the complex in CH_2_Cl_2_ layered with *n*-pentane. ^1^H NMR (δ, CD_2_Cl_2_, 20 °C):5.77 (s, 1H, Pyr^3,4^), 5.64 (s, 1H, Pyr^3,4^), 4.11 (bs, 1H, CH_2_), 3.19 (d, *J* = 7.6 Hz, 1H, CH_2_), 2.87 (d, *J* = 10.5 Hz, 2H, CH_2_), 2.78–2.61 (m, 2H, CH_3_C*H*CH_3_), 2.54–2.45 (m, 2H, CH_3_C*H*CH_3_), 2.32 (bs, 2H, CC*H*_2_C*H*_2_C*H*_2_C*H*_2_), 2.12 (bs, 2H, CC*H*_2_C*H*_2_C*H*_2_C*H*_2_), 1.94 (bs, 2H, CC*H*_2_C*H*_2_C*H*_2_C*H*_2_), 1.88 (bs, 2H, CC*H*_2_C*H*_2_C*H*_2_C*H*_2_), 1.56–1.18 (m, 24H, C*H*_3_CHC*H*_3_). ^13^C NMR (δ, CD_2_Cl_2_, 20 °C): 141.2 (vdd, *J* = 8.2, 3.9 Hz, Pyr^2,5^), 126.4 (vd, *J* = 11.6 Hz, Pyr^2,5^), 111.8 (vd, *J* = 8.5 Hz, Pyr^3,4^), 103.3 (vdd, *J* = 7.1, 2.5 Hz, Pyr^3,4^), 98.2 (vdd, *J* = 48.3, 8.3 Hz, CH_2_*C*CH_2_), 39.0 (C*C*H_2_*C*H_2_*C*H_2_*C*H_2_), 26.4 (vd, *J* = 17.4 Hz, CH_2_), 25.9 (d, *J* = 8.7 Hz, CH_3_*C*CH_3_), 24.0 (C*C*H_2_*C*H_2_*C*H_2_*C*H_2_), 23.9 (C*C*H_2_*C*H_2_*C*H_2_*C*H_2_), 23.7 (C*C*H_2_*C*H_2_*C*H_2_*C*H_2_), 23.4 (C*C*H_2_*C*H_2_*C*H_2_*C*H_2_, CH_3_*C*CH_3_), 23.0 (C*C*H_2_*C*H_2_*C*H_2_*C*H_2_), 19.8 (vd, *J* = 11.4 Hz, CH*C*H_3_), 18.0 (vd, *J* = 3.7 Hz, CH*C*H_3_), 17.7 (vd, *J* = 3.5 Hz, CH*C*H_3_). ^31^P{^1^H} NMR (δ, CD_2_Cl_2_, 20 °C): 35.4, 31.8. Anal. Calcd for C_23_H_42_Cl_3_NOP_2_Ti: C, 48.92; H, 7.50; N, 2.48. Found: C, 49.15; H, 7.32; N, 2.38.

### X-ray Structure Determination

X-ray diffraction data of **2**–**5** (CCDC 2283445–2283448) were collected at *T* = 100 K in a dry stream of nitrogen on a Bruker Kappa APEX II diffractometer system using graphite-monochromatized Mo-*K*α radiation (λ = 0.71073 Å) and fine-sliced φ- and ω-scans. Data were reduced to intensity values with SAINT, and an absorption correction was applied with the multiscan approach implemented in SADABS.^13^ The structures were solved by the dual-space approach implemented in SHELXT^14^ and refined against *F*^2^ with SHELXL.^15^ Non-hydrogen atoms were refined with anisotropic displacement parameters. H atoms were placed in calculated positions and thereafter refined as riding on the parent atoms. Molecular graphics were generated with the program MERCURY.^16^

### Computational Details

The computational results presented have been achieved in part using the Vienna Scientific Cluster (VSC). All calculations were performed using the Gaussian 09 software package^17^ without symmetry constraints. The optimized geometries were obtained with the PBE0 functional. That functional uses a hybrid generalized gradient approximation (GGA), including a 25% mixture of Hartree–Fock^18^ exchange with DFT^19^ exchange-correlation, given by Perdew, Burke, and Ernzerhof functional (PBE).^20^ The basis set used consisted of the Stuttgart/Dresden ECP (SDD) basis set^21^ to describe the electrons of titanium, and a standard 6-31G(d,p) basis set^22^ for all other atoms. Transition state optimizations were performed with the Synchronous Transit-Guided Quasi-Newton Method (STQN) developed by Schlegel and co-workers,^23^ following extensive searches of the potential energy surface. Frequency calculations were performed to confirm the nature of the stationary points, yielding one imaginary frequency for the transition states and none for the minima. Each transition state was further confirmed by following its vibrational mode downhill on both sides and obtaining the minima presented on the energy profiles. The electronic energies were converted to free energy at 298.15 K and 1 atm by using zero-point energy and thermal energy corrections based on structural and vibration frequency data calculated at the same level.

## Supporting Information Available

^1^H, ^13^C{^1^H}, and ^31^P{H} NMR spectra of all compounds (PDF)
Cartesian coordinates for DFT-optimized structures (XYZ)

## Supplementary Material

om3c00327_si_001.pdf

om3c00327_si_002.xyz
