# Supplementary material for: Pyrrole-Based Ti(III) and Ti(IV) PNP Pincer Complexes: Insertion of Ketones into the Ti(IV)-Phosphorus Bond
Source: Organometallics. 2023 Oct 5;42(20):2999–3004. doi: 10.1021/acs.organomet.3c00327 (PMC10598877; doi:10.1021/acs.organomet.3c00327)
Supplement: Supplementary file 1 — om3c00327_si_001.pdf [file om3c00327_si_001.pdf]

Pyrrole-based Ti(III) and Ti(IV) PNP Pincer Complexes: Insertion of Ketones into the Ti(IV)-Phosphorous Bond

Gerald Tomsu,[†] Berthold Stöger,[‡] Karl Kirchner*[,†]

[†] Institute of Applied Synthetic Chemistry, TU Wien, Getreidemarkt 9/163-AC, A-1060 Wien, Austria

[‡] X-Ray Center, TU Wien, Getreidemarkt 9/163, A-1060 Wien, Austria

Supporting Information

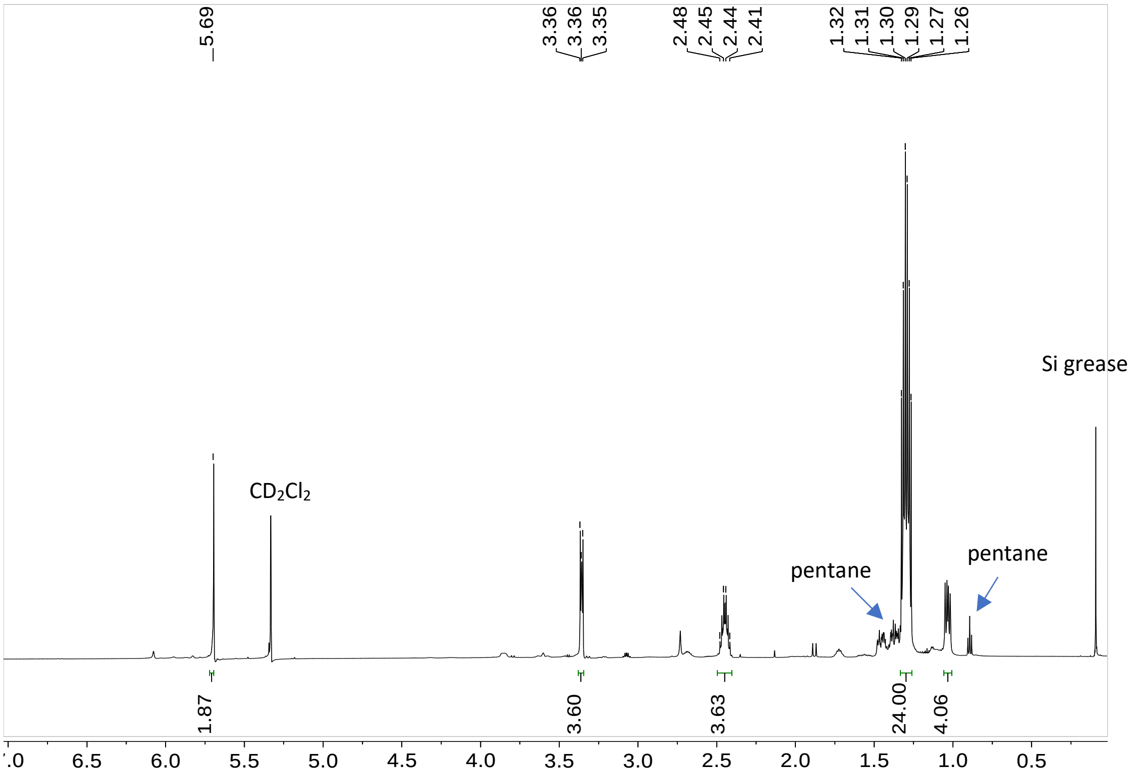

Figure S1. ^{1}**H NMR Spectrum of [Ti(PNP-*i*Pr)(Cl)$_3$] (2)**

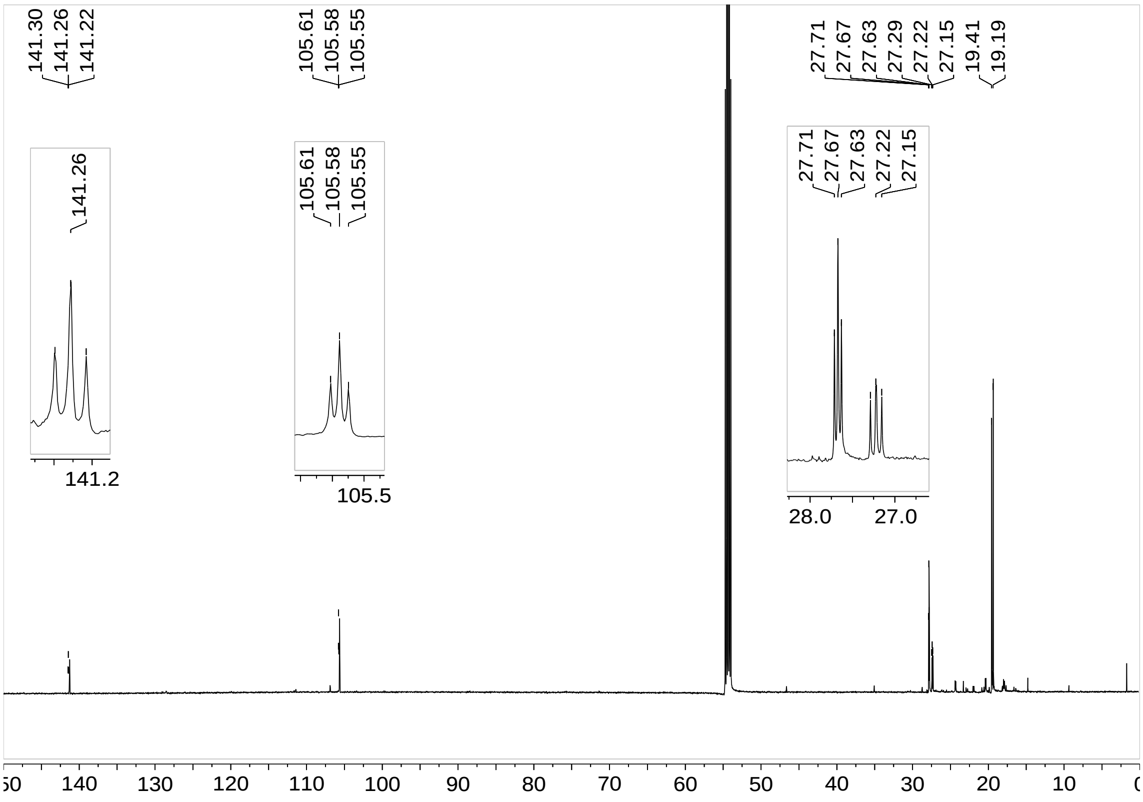

Figure S2. ^{13}**C{**^{1}**H} NMR Spectrum of [Ti(PNP-*i*Pr)(Cl)$_3$] (2)**

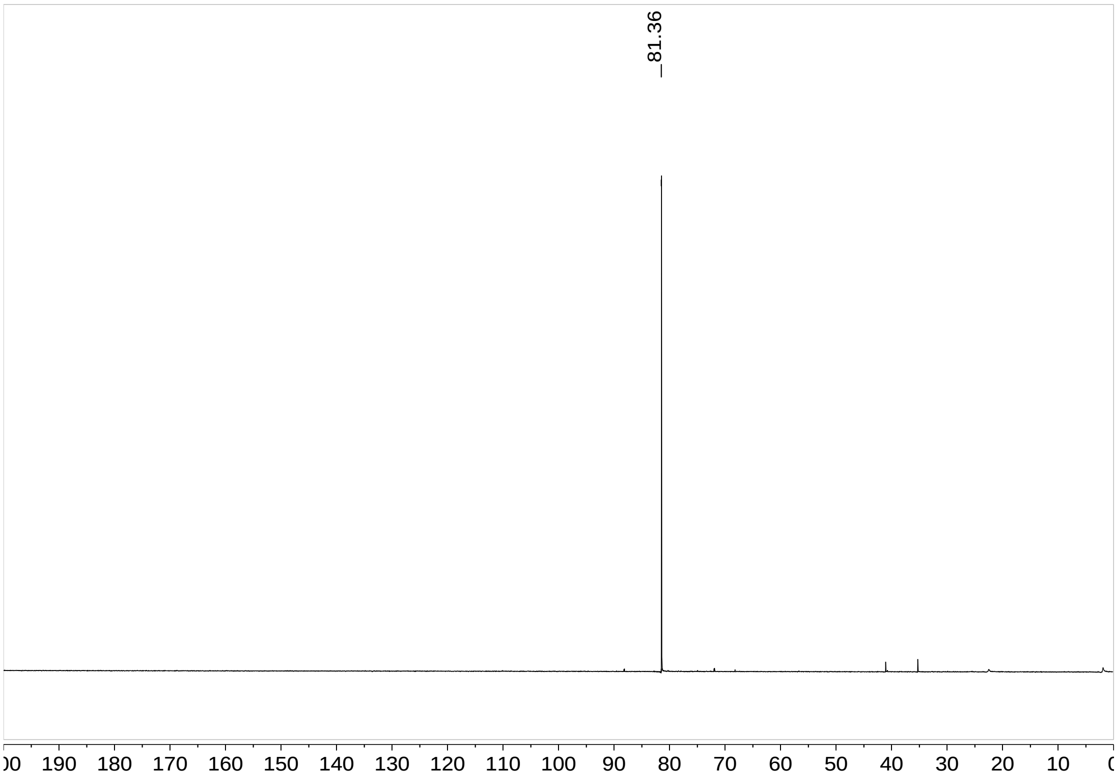

Figure S3. $^{31}P\{^{1}H\}$ NMR Spectrum of [Ti(PNP-*i*Pr)(Cl)$_3$] (2)

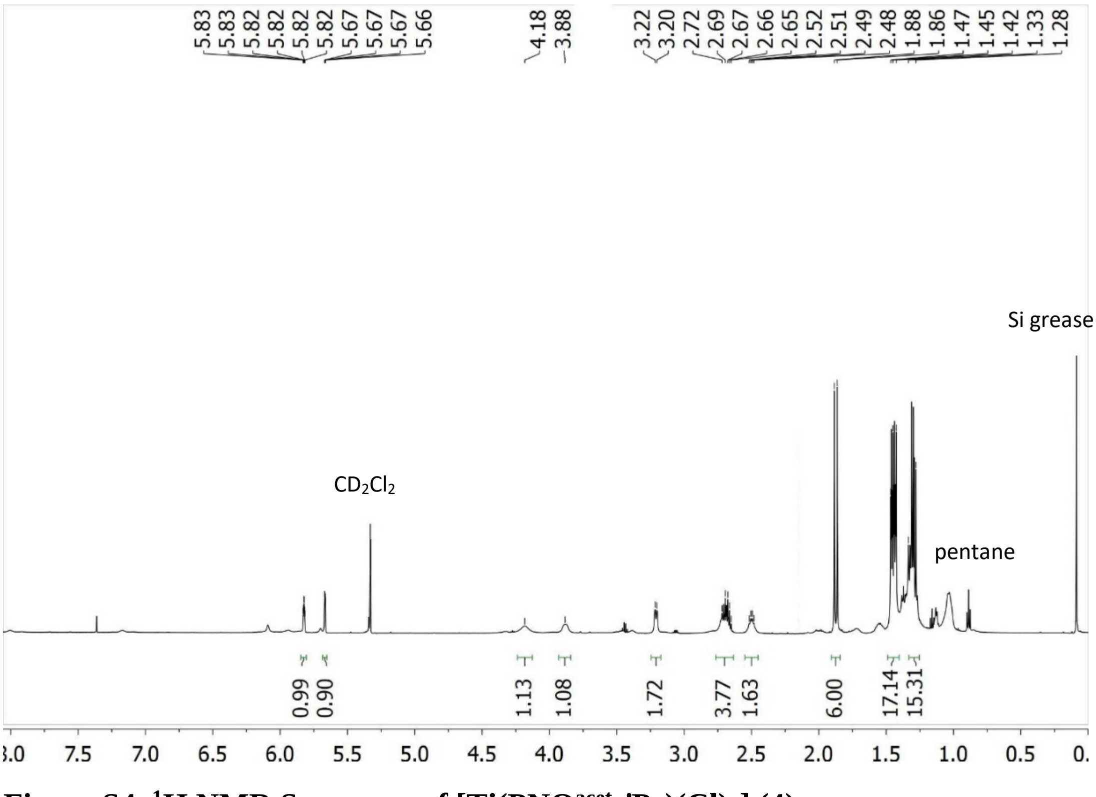

Figure S4. ^{1}H NMR Spectrum of [Ti(PNO^{acet}-*i*Pr)(Cl)$_3$] (4)

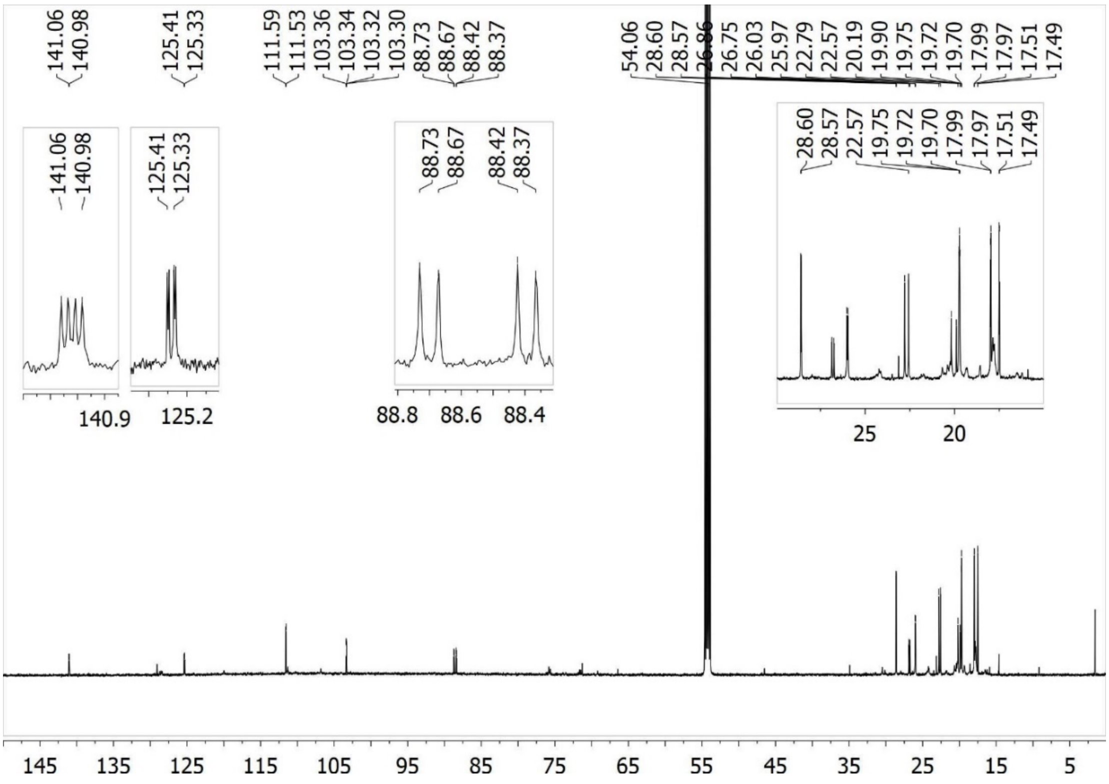

Figure S5. $^{13}C\{^{1}H\}$ **NMR Spectrum of [Ti(PNO^{acet}-*i*Pr)(Cl)$_3$] (4)**

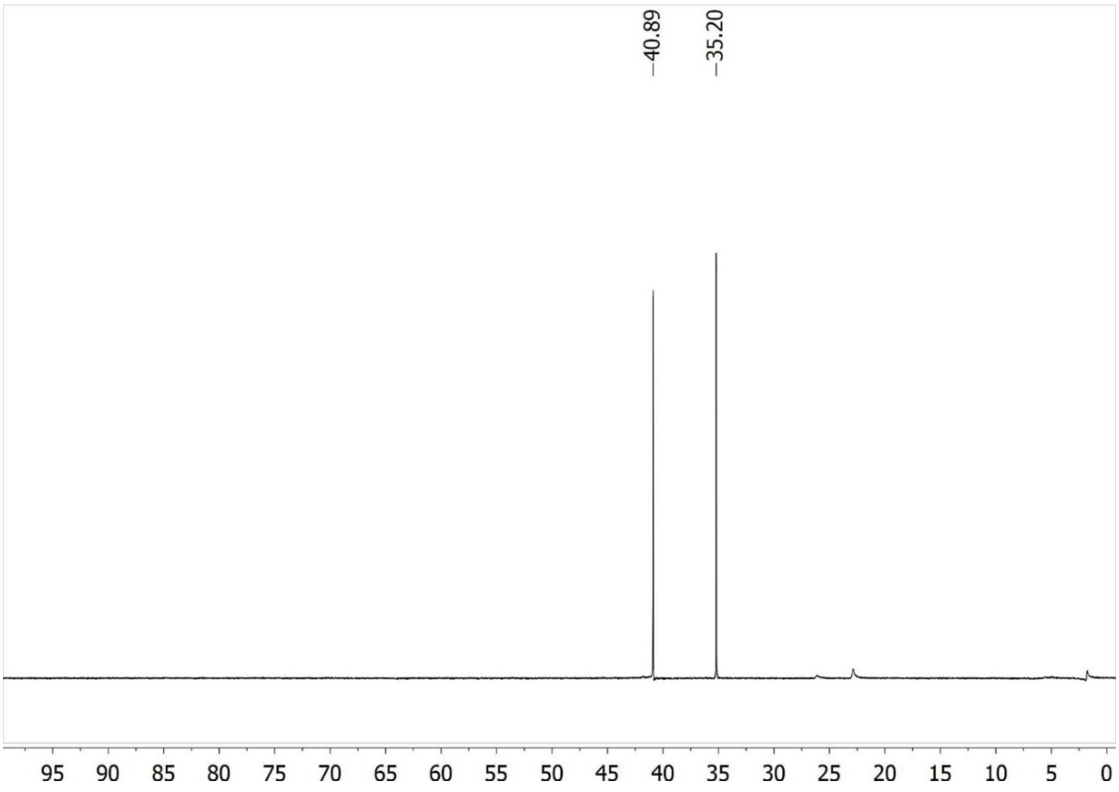

Figure S6. $^{31}P\{^{1}H\}$ **NMR Spectrum of [Ti(PNO^{acet}-*i*Pr)(Cl)$_3$] (4)**

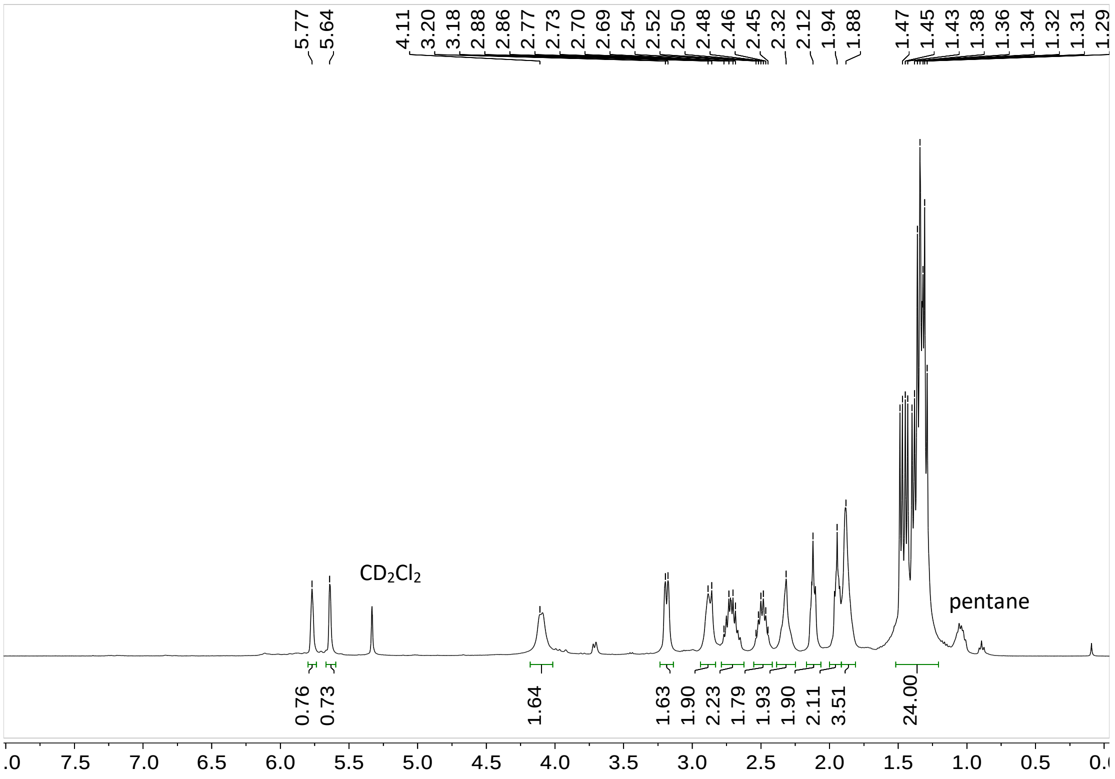

Figure S7. ^{1}H NMR Spectrum of [Ti(PNO^{cyclo}-*i*Pr)(Cl)$_3$] (5)

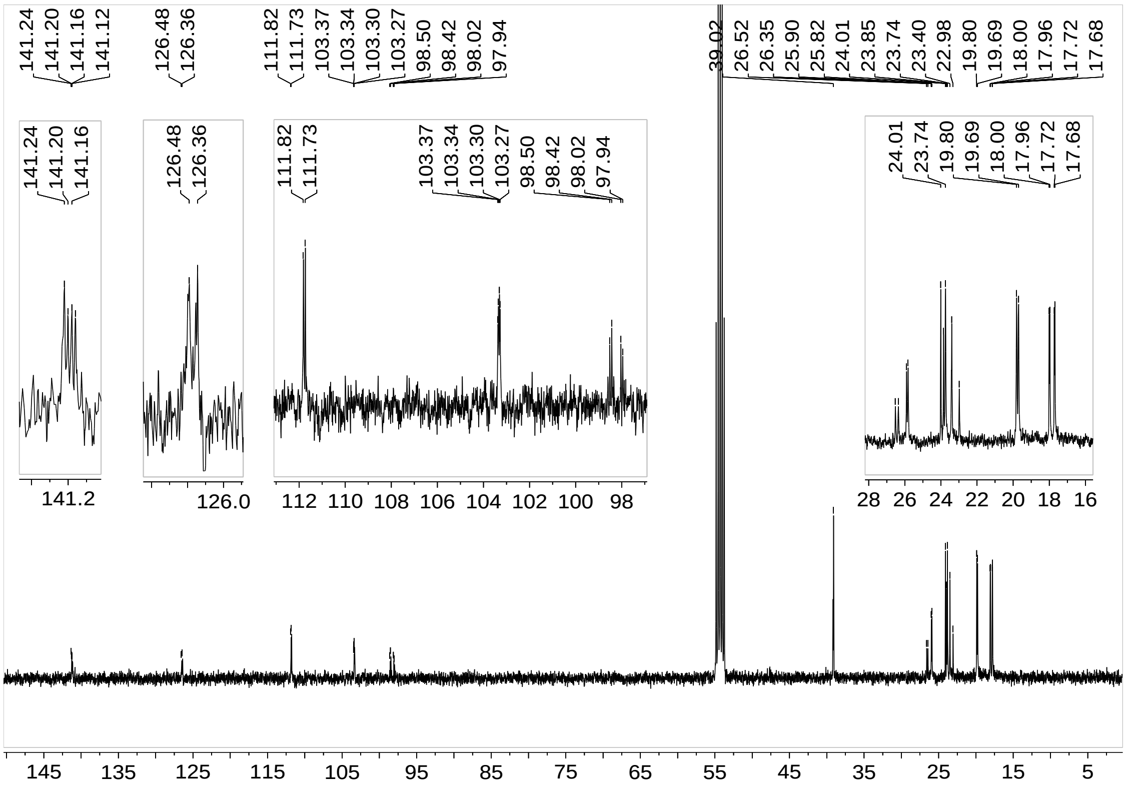

Figure S8. ^{13}C{^{1}H} NMR Spectrum of [Ti(PNO^{cyclo}-*i*Pr)(Cl)$_3$] (5)

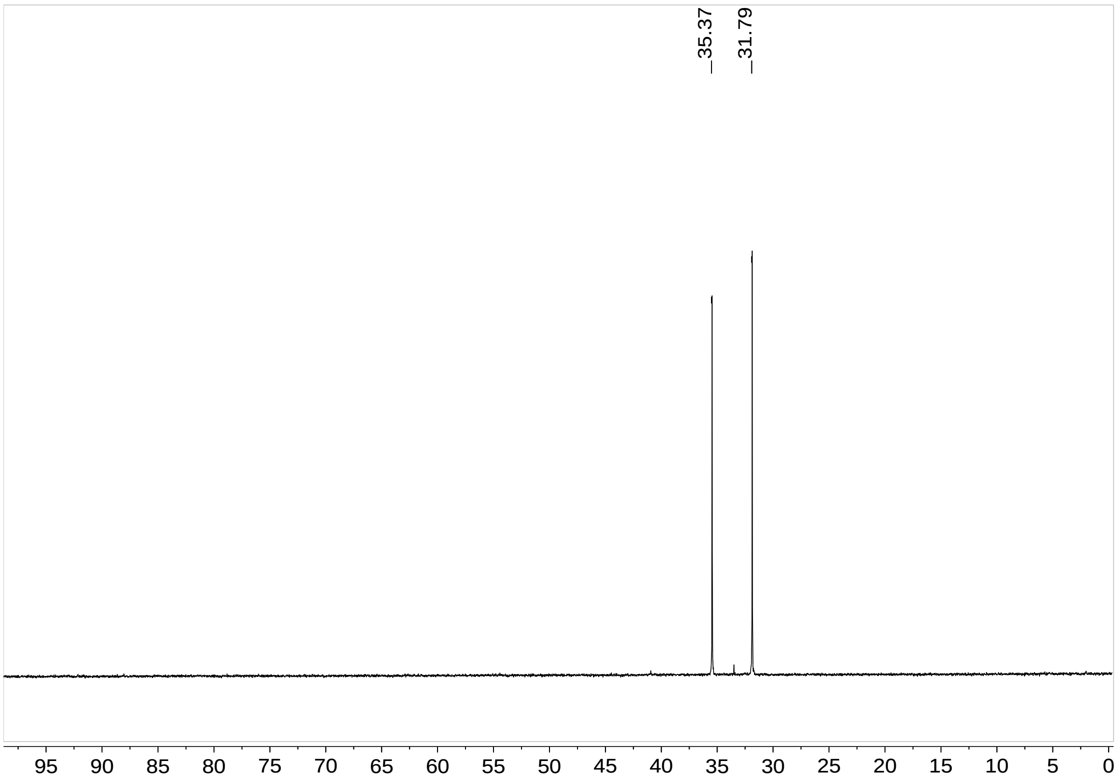

Figure S9. $^{31}P\{^{1}H\}$ **NMR Spectrum of [Ti(PNO^{cyclo}-*i*Pr)(Cl)$_3$] (5)**
